# Transvaginal double-layer parallel in-situ suturing for early complex vesicovaginal fistula repair: Case report

**DOI:** 10.1097/MD.0000000000039881

**Published:** 2024-10-11

**Authors:** Chuanfeng Liu, Shouxia Cao, Haiyan Liu, Qingtan Pang, Zichao Zhao, Fuming Wang, Yongqiang Xia

**Affiliations:** a Department of Urology, Linyi Maternity and Child Health Care Hospital, Shandong Province, China; b Clinical Medicine Department, Shandong Medical College, Shandong Province, China; c Department of Anesthesiology, Linyi Maternity and Child Health Care Hospital, Shandong Province, China.

**Keywords:** complex vesicovaginal fistula, early repair, transvaginal repair technique

## Abstract

**Rationale:**

Complex vesicovaginal fistulas (VVFs) with large defects pose significant surgical challenges. Traditional repair methods often require extensive tissue separation and multilayer suturing, risking local blood supply and healing. This study introduces a novel modified transvaginal repair technique that simplifies the procedure while preserving tissue vascularity. It employs double-layer parallel in situ suturing for early repair of complex VVF.

**Patient concerns:**

A 50-year-old woman was admitted with continuous vaginal urine leakage for 4 days following trauma. Speculum examination revealed a 3-cm longitudinal oval laceration at the 11 o’clock position in the dorsal lithotomy site, with continuous fluid leakage through the fistula.

**Diagnoses:**

Self-inflicted complex VVF.

**Interventions:**

The patient underwent prophylactic placement of bilateral double-J stents and continuous catheterization, followed by surgical repair using a modified transvaginal technique involving double-layer parallel in situ suturing.

**Outcomes:**

Postoperative evaluations showed successful healing with no urinary leakage. The vaginal sutures were removed on day 24, and follow-up at 1 year confirmed no recurrence of the fistula or lower urinary tract symptoms, significantly improving the patient’s quality of life.

**Lessons:**

The modified transvaginal repair technique using double-layer parallel in situ suturing is a simple and effective approach for early repair of complex VVF, highlighting its potential for broader clinical application. Future studies with larger cohorts are needed to validate these findings.

## 1. Introduction

Vesicovaginal fistula (VVF) is an abnormal anatomical connection between the bladder and vagina, and it is a common type of female urogenital tract fistula. With the improvement of obstetric surgical techniques and the increase in minimally invasive gynecological surgeries in some developing countries, the proportion of VVFs due to gynecological surgeries has surpassed other causes.^[1,2]^ VVF significantly impairs lower urinary and sexual functions, leading to substantial mental and psychological stress, thereby severely affecting patients’ health-related quality of life.^[3]^

Surgery is the primary treatment modality. Surgical approaches include transvaginal, transvesical, transabdominal, laparoscopic, and robotic pathways.^[4]^ Since Latzko’s procedure was first implemented over 80 years ago,^[5]^ surgical techniques for VVF have continuously evolved and been refined due to the characteristics of different fistulas and physician preferences. However, the transvaginal approach remains generally preferred.^[6]^ Despite advancements, straightforward surgical methods for complex VVF with larger fistulas are lacking. For complex VVF with larger fistulas, we introduce a modified transvaginal repair technique that requires only 2 layers of parallel in situ suturing, eliminating the need for traditional perpendicular suturing.^[7]^ This approach reduces the separation area around the fistula, minimizes surgical trauma, and effectively preserves tissue blood supply, as illustrated by the successful treatment of a patient with a complex VVF.

## 2. Case presentation

A 50-year-old female patient with a BMI of 30.8 kg/m² and a known history of mild mental disorder presented to the hospital with persistent vaginal leakage for 4 days following trauma. On June 5, 2022, under the suspicion of abdominal fluid accumulation, she manipulated and pulled the anterior vaginal wall post-urination, subsequently cutting parts of both the vaginal wall and bladder using scissors. Initially, the injury resulted in a flow of fresh red blood, which transitioned into a continuous stream of lighter red fluid over time. Since the self-inflicted incident, the patient had been unable to urinate independently. Her medical history included a cesarean delivery in 2008, with no other significant diseases reported. A speculum examination revealed a 3 cm longitudinal oval laceration at the 11 o’clock position of the dorsal lithotomy site, through which fluid introduced via a catheter was continuously leaking vaginally. The surrounding tissue exhibited mild inflammation without active bleeding, thereby confirming the diagnosis of a self-inflicted VVF.

**Figure 1.**
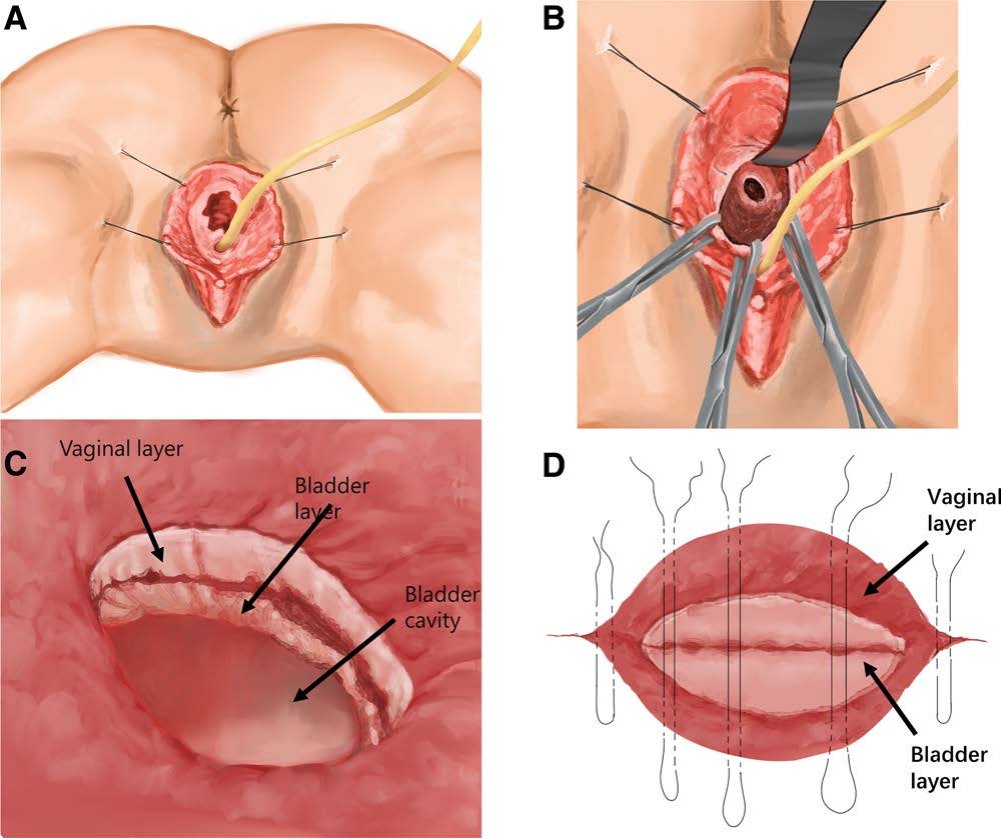
Transvaginal VVF repair process. (A) Exposing the vaginal vestibule. (B) Exposing the fistula. (C) Separating the bladder and vaginal walls. (D) In situ suturing of the bladder and vaginal walls. VVF = vesicovaginal fistula.

**Figure 2.**
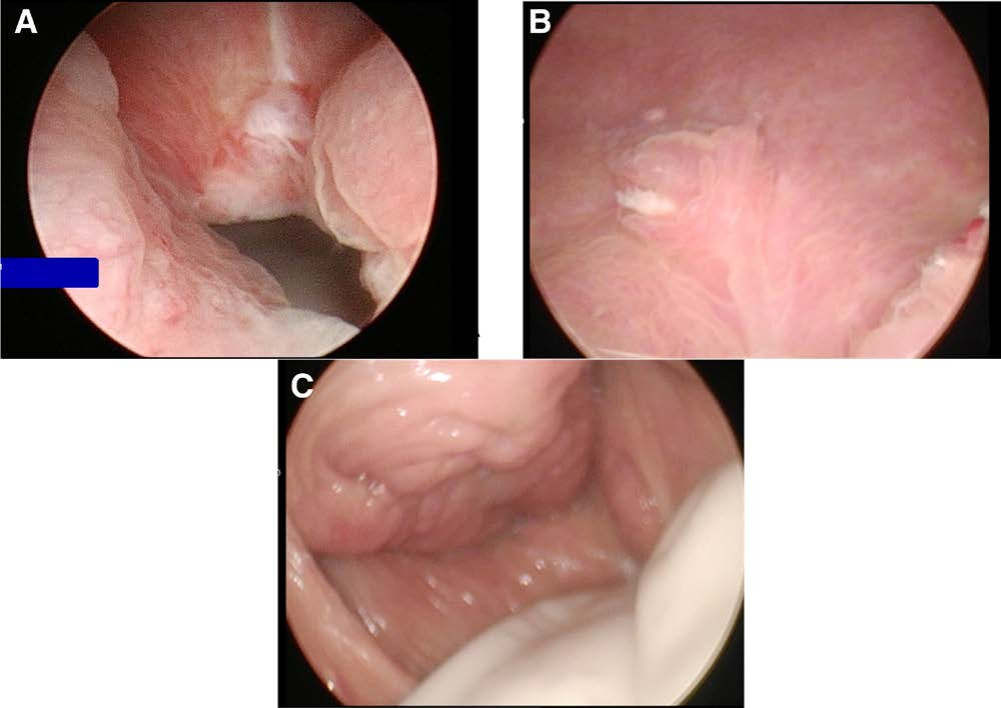
Endoscopic findings before and after surgery. (A) The fistula is clearly visible, extending from the interureteric ridge to the posterior wall of the bladder, longitudinally oval in shape, approximately 3cm in length. (B) The fistula has healed. (C) Vaginoscopy reveals healing of the fistula.

The patient underwent combined spinal and epidural anesthesia and was initially positioned in a dorsal lithotomy position. Cystoscopy identified bilateral ureteral orifices in their normal positions with discernible urine flow. A longitudinal oval laceration, approximately 3 cm in length, was noted above the interureteric ridge on the left side, with the laceration’s edge situated about 2 cm from the left ureteral orifice and trajectory area (Fig. 2A). To avert ureteral damage during the procedure, bilateral 5Fr ureteral Double-J stents were placed, and a decision was made to proceed with a transvaginal VVF repair. The surgical steps included: Exposing the vaginal vestibule: The patient was repositioned in a prone position with legs apart. The labia minora were sutured and secured, and an 18Fr catheter with a 10 mL water-filled balloon was inserted (Fig. 1A); Exposing the fistula: Retractor hooks were employed to separate the posterior and lateral walls of the vagina, and Allis forceps were used to clamp the edges on both sides and the upper edge of the fistula, thereby exposing the operative field (Fig. 1B); Separating the bladder and vaginal walls: An appropriate volume of saline was injected into the tissue between the bladder and vagina around the fistula to expand the interstitial space. A circumferential incision was then executed around the fistula using an electrosurgical unit, and vascular clamps were utilized to separate the bladder and vaginal walls, maintaining a depth of approximately 1 cm from the fistula edge (Fig. 1C); Suturing the bladder wall: The bladder wall was sutured longitudinally with 3-0 absorbable sutures, ensuring that the suture edges were inverted into the bladder as much as possible. The ends of the sutures were concealed within the upper and lower ends of the tissue between the bladder and vagina. Fine instruments were used to invert the bladder mucosal tissue along the suture line into the bladder cavity (Fig. 1D). Bladder irrigation via catheter indicated no leaks; Suturing the vaginal wall: 2-0 absorbable sutures were used to perform intermittent vertical mattress suturing of the vaginal wall parallel to the direction of the bladder. During suturing, it was ensured that no dead spaces existed between the bladder and vaginal wall tissues, and the knotting tension was adjusted to significantly evert the vaginal wall tissue, minimizing contact between the vaginal wall sutures and the bladder wall sutures. Following the completion of suturing, water was injected into the bladder through a catheter to verify the absence of leakage (Fig. 1D). The surgery concluded with the insertion of iodine-soaked gauze into the vagina for hemostatic compression.

Postoperatively, the patient was administered antibiotics for a duration of 9 days. On the 24th postoperative day, the vaginal sutures were extracted. On the 27th postoperative day, a cystoscopy performed under urethral mucosal anesthesia demonstrated the healing of the fistula on the bladder side (Fig. 2B). A subsequent vaginoscopy confirmed the adequate healing of the vaginal wall (Fig. 2C). The administration of methylene blue solution via the catheter resulted in no dye being detected in the vagina, thereby confirming the successful repair of the VVF. The catheter was then removed, and the patient gradually regained normal urination capabilities. At the 1-year follow-up, the patient showed no signs of fistula recurrence and no symptoms related to the lower urinary tract. The patient provided written informed consent for the publication of this case report, as approved by the Ethics Committee of Linyi Maternity and Child Health Care Hospital.

## 3. Discussion

The modified transvaginal repair technique using double-layer parallel in situ suturing was successfully applied to a 50-year-old female patient with a complex VVF. The patient showed rapid postoperative recovery, with no complications such as urinary leakage or infection, and resumed normal urination shortly after catheter removal. Long-term follow-up over 1 year demonstrated no recurrence of the fistula and no lower urinary tract symptoms, significantly improving the patient’s quality of life. These favorable outcomes indicate that this technique can reduce surgical trauma, maintain adequate blood supply, and avoid complications associated with more invasive procedures. The simplicity and effectiveness of this method highlight its potential for broader clinical application, especially in scenarios where traditional perpendicular suturing methods may not be feasible.

The management of VVF encompasses both conservative and surgical modalities. Conservative treatments typically involve indwelling catheterization for a specified duration, electrocoagulation, and the administration of blood-derived products such as platelet-rich plasma and fibrin glue.^[6,8,9]^ Contemporary surgical repair techniques are predominantly informed by expert opinions and case series reports, thereby lacking a standardized consensus on the surgical approach. Surgical interventions for VVF include transvaginal, transvesical, transabdominal, laparoscopic, and robotic routes,^[4]^ with the selection of tissue interposition contingent upon intraoperative conditions. The transvaginal approach is applicable to the majority of VVF cases and is generally favored.^[4,6]^ Relative to abdominal approaches, the transvaginal method is associated with reduced blood loss, fewer complications, diminished patient stress, and abbreviated hospital stays.^[10]^ In comparison to minimally invasive techniques (e.g., laparoscopic or robotic routes), the transvaginal approach boasts shorter surgical and hospitalization durations, decreased blood loss, lower costs, and elevated success rates.^[11]^ Transabdominal or transvesical approaches may be utilized for fistulas situated in challenging-to-access regions within the vaginal vault or in instances necessitating concurrent abdominal surgery.^[12]^ Vascularized flaps are frequently deployed in the repair of complex VVF cases, particularly those associated with pelvic irradiation or recurrent fistulas.^[6]^

Vesicovaginal fistulas (VVFs) are categorized into various types, typically classified as either simple or complex. In the present case, the VVF is designated as complex, primarily due to its diameter, which exceeds 2.5 cm. Complex VVFs encompass those arising from chronic diseases, recurrent fistulas subsequent to previous repair surgeries, and those induced by radiation therapy or pelvic malignancies.^[13]^

**Figure 3.**
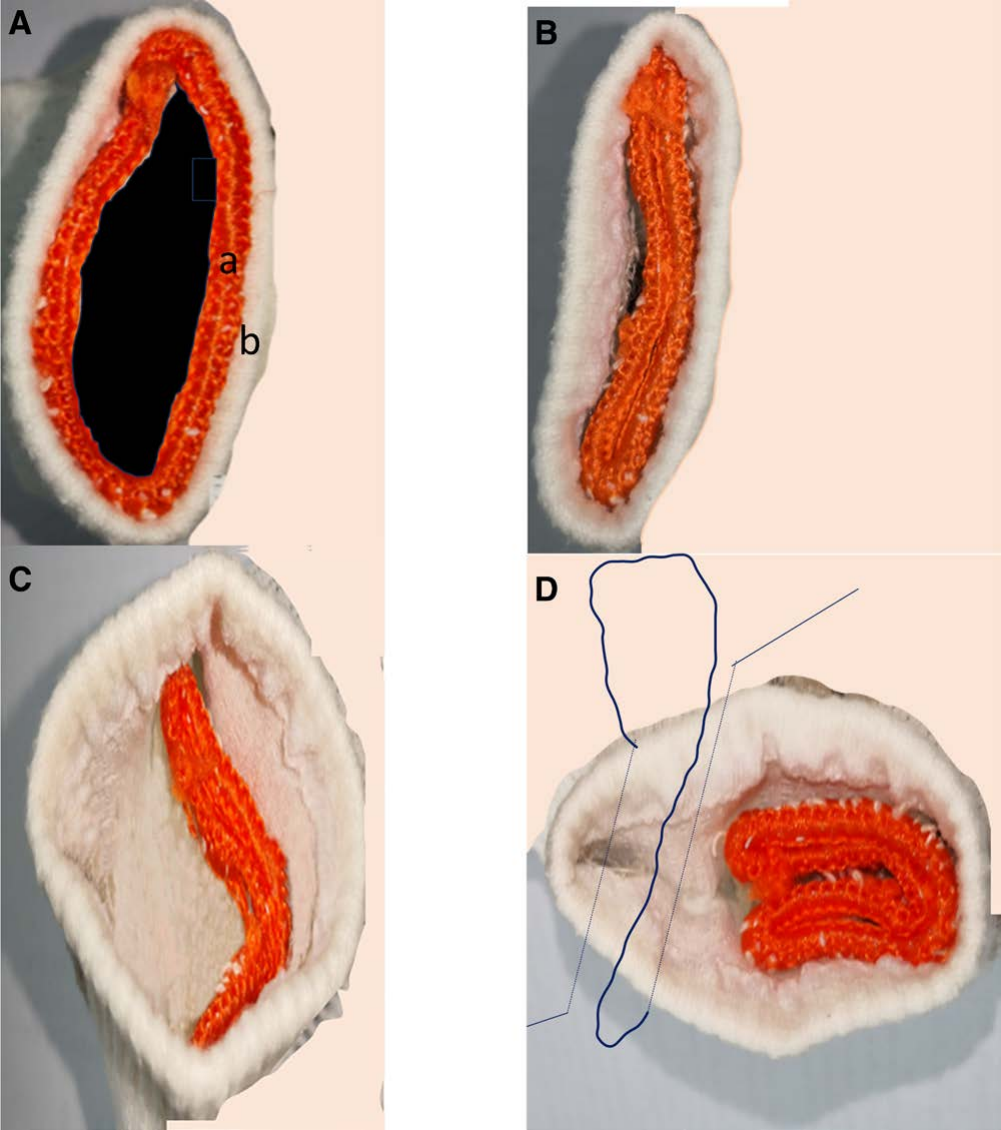
Technical status when the separation area is insufficient before technical improvement. (A) Preoperative condition of the fistula; a represents the bladder wall of the fistula, b represents the vaginal wall of the fistula. (B) Status after suturing the bladder wall of the fistula. (C) State intended for perpendicular suturing of vaginal wall with bladder tissue after deep separation between the vaginal wall and bladder wall of the fistula. (D) State just before completion of perpendicular suturing between vaginal wall and bladder wall.

Latzko’s technique, one of the most extensively utilized surgical methods, primarily entails the circumferential mobilization of the vaginal mucosa surrounding the fistula, followed by the layered closure of the pubovesical fascia and vaginal mucosa. Subsequent reports often integrate enhancements based on individual surgical experiences, frequently involving 3-layer or multi-layer suturing, with a focus on non-overlapping sutures.^[5,10,11,14–18]^ A perpendicular approach is commonly adopted to prevent the overlap of sutures.^[7,17,19,20]^ In clinical practice, our observations indicate that when the suture lines of the vaginal wall and the bladder are perpendicular, a substantial separation area between the vaginal and bladder walls is required. An inadequate separation area can lead to the folding of the bladder wall along the suture lines post-suturing, resulting in suture accumulation and compromised blood circulation, which is unfavorable for fistula healing (Fig. 3). Conversely, when there is extensive mobilization of the area between the vaginal and bladder walls: notwithstanding the limitations of the surgical field: it intensifies the difficulty and trauma of the procedure, potentially culminating in a residual dead space following suturing, which similarly hinders fistula healing. To address this issue, we have implemented technical advancements concerning the separation area: an appropriate separation area between the vaginal wall and bladder wall at the fistula site, suturing both the full layers of the bladder wall and anterior vaginal wall in 2 layers with a parallel orientation. For the bladder wall, we utilized 3-0 absorbable sutures for continuous intracorporeal suturing, whereas for the vaginal wall, we employed 2–0 absorbable sutures for interrupted vertical mattress suturing. Moderate suture tension aids in eliminating potential dead spaces between the bladder and vaginal wall tissues and diminishes the contact between sutures on the vaginal and bladder walls. Through these technical refinements, double-layer parallel suturing accomplishes the objective of non-overlapping suture lines, precluding the accumulation of foreign bodies within the fistula that may transpire when suturing layers exceed 3; a moderate separation area between the vaginal and bladder walls safeguards local blood circulation, mitigating the risk of fistula non-healing.

During the surgical procedure, we observed that the tissue surrounding the fistula exhibited minimal inflammation and was rich in blood vessels. Given the absence of a history of malignancy or radiation therapy, no flap intervention was deemed necessary. We did not encounter high-quality evidence advocating for the routine use of flap interposition. The operation was predicated on airtight, tension-free, and infection-free parallel double-layer suturing, which proved adequate. Furthermore, we administered saline into the space between the bladder and vaginal tissues around the fistula to augment the depth of the fistula tissues. At the juncture between the bladder and vaginal wall, we employed an electrosurgical unit to execute a circumferential incision, facilitating precise separation of 1 cm around the fistula for hemostasis and the excision of unhealthy tissues. No trimming of the fistula tissue was conducted to avert enlarging the fistula, thereby facilitating in situ healing of the vitalized tissue surface. Min Tang and colleagues^[12]^ managed 57 VVF patients utilizing a technique that involved single-layer suturing with a circular vaginal flap, attaining a high 1-time success rate of 82.3% via a vaginal approach. However, we found that most cases were simple VVFs, with the fistula being left untreated and opening into the bladder, posing a potential risk of bladder wall bleeding. In light of our center’s experience, the technique of parallel in situ double-layer suturing is comparatively straightforward for complex fistulas with a diameter of 3 cm, ensuring the preservation of blood circulation while maintaining acceptable tissue tension. Consequently, VVF repair surgery via the vaginal approach is deemed feasible.

We conducted the surgery on the fifth day post-trauma, marking a bold endeavor. The optimal timing for surgery remains a subject of debate: while many experts advocate for repair within 72 hours of trauma, in practice, most fistulas are identified several days to weeks post-injury, frequently requiring a postponement of 3 to 6 months for surgical intervention^.[2,21]^ Early repair is favored when the wound tissue is still fresh, whereas late repair is employed to allow inflammation to recede and to prevent infection. For fistulas arising from gynecological surgery, it is recommended to defer surgical repair for 8 to 12 weeks, irrespective of the condition of the surrounding tissue, to permit inflammation to diminish and surgical sutures to be absorbed.^[11]^ However, we contend that the timing for VVF repair ought to be dictated by the specific circumstances of the fistula. The failure of VVF repair is frequently associated with acute inflammation, edema, ischemia, or necrosis; hence, the decision on repair timing hinges on whether necrotic tissues and inflammatory alterations can be effectively addressed.^[22]^ We observed that the devised transvaginal parallel double-layer VVF repair technique presents a lower degree of difficulty, superiorly safeguards blood circulation, and is tolerant of mild inflammation around the fistula in this instance, thereby challenging conventional timing recommendations for transvaginal VVF repair and enabling early intervention. Humanistic care is also a factor^[23]^; the patient in this case faces mental health challenges, is challenging to care for, and the persistent incontinence significantly affects family life. The patient and her family members are urgently seeking resolution to these issues, with the goal of a rapid return to normalcy. In conclusion, the selection of the timing for surgery is influenced by a multitude of factors. Deciding to perform the surgery 5 days post-trauma was a viable and correct decision.

During the dressing change, increased vaginal discharge was noted, and the 2-0 absorbable suture retained significant tension and might not be absorbed shortly. Considering the potential for linear fistula formation, we opted to remove the sutures before catheter removal to mitigate the risk of such complications. This intervention aims to safeguard the surgical site’s healing process, preventing new issues caused by excessive suture tension.

While this study introduces a novel transvaginal parallel double-layer in situ suturing technique for the repair of complex VVF, it is essential to acknowledge some limitations. First, the findings are based on a single case report, which limits the generalizability of the results. The technique’s efficacy and safety have not been validated in larger patient populations or through prospective longitudinal studies, which are necessary to establish its broader applicability. Moreover, while this technique may be feasible and beneficial in straightforward cases, its effectiveness in more complicated cases involving radiation-induced fistulas, recurrent fistulas, or extensive fibrosis has not been evaluated. These limitations highlight the need for further research with larger sample sizes and a more diverse patient population to understand this approach’s potential advantages and constraints comprehensively.

## 4. Conclusion

We initiated a novel transvaginal parallel double-layer in situ suturing technique, successfully repairing a complex VVF case. During the procedure, circumferential electrocautery of the fistula was performed to eliminate or reduce fistula scarring. A circumferential area of 1 cm depth was dissected, making separation and suturing technically easier with less trauma, thus preserving local blood circulation. The fistula was not trimmed, avoiding increased suture tension. Timely removal of the 2-0 absorbable vaginal sutures was beneficial in preventing the formation of linear fistulas and ensuring smooth healing. This surgical procedure is characterized by its simplicity, low technical difficulty, and ease of learning and dissemination. There was no extensive circumferential separation of the fistula during the operation, and the procedure remained in situ, allowing for secondary transvaginal repair in case of initial VVF repair failure. However, it is important to note that this innovation is presently a case report, lacking prospective longitudinal studies and larger sample sizes to substantiate its academic value, thereby necessitating further exploration.

## Author contributions

**Conceptualization:** Chuanfeng Liu.

**Data curation:** Shouxia Cao, Fuming Wang.

**Formal analysis:** Shouxia Cao.

**Investigation:** Zichao Zhao.

**Methodology:** Yongqiang Xia.

**Resources:** Haiyan Liu.

**Software:** Shouxia Cao, Qingtan Pang.

**Supervision:** Zichao Zhao, Fuming Wang.

**Visualization:** Qingtan Pang.

**Writing – original draft:** Chuanfeng Liu.

**Writing – review & editing:** Yongqiang Xia.
